# Supplementary material for: There Is Strength in Numbers: Quantitation of Fc Gamma Receptors on Murine Tissue-Resident Macrophages
Source: Int J Mol Sci. 2021 Nov 10;22(22):12172. doi: 10.3390/ijms222212172 (PMC8620503; doi:10.3390/ijms222212172)
Supplement: Supplementary file 1 [file ijms-22-12172-s001.zip › Supplementary Methods S1- Preparation of Tissue Macrophages - revised reference numbering.pdf]

Methods S1

Preparation of Tissue-Resident Macrophages

Preparation of Alveolar and Interstital Macrophages from mouse lung. 2

Preparation of macrophages from mouse spleen 4

Preparation of Langerhans cells and Dermal Macrophages from mouse skin (ears) 6

Preparation of Kupffer cells from mouse liver... 8

Preparation of murine Kidney-resident Macrophages.. 9

Preparation of microglia from mouse brain 11

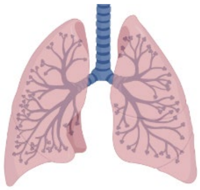

Preparation of Alveolar and Interstitial Macrophages from mouse lung.

(Adapted from a protocol by Cossarizza et al. [76])

Broncho-alveolar lavage (BAL)

(Adapted from a protocol by Van Hoeke et al. [77])

Mice were euthanized without cranial dislocation to avoid tearing of the trachea. Subsequently, the pulmonary circulation was perfused with at least 3 ml PBS via the heart. This step is preparatory for enzymatic digestion of the lung after BAL. The trachea was exposed by performing a small incision near the larynx and inserting a catheter with a 20G needle. By using a 2ml syringe, ~1ml of PBS was slowly injected through the catheter until the lung expanded to a normal volume and was filling the chest captivity. During this step it is important to ensure that the lung does not get hyperinflated to avoid tissue rupture. Subsequently the fluid was re-aspirated and transferred to a 15 ml conical tube. The flushing and re-aspiration was then repeated 3-5 times while using fresh PBS each time. The lavage samples were then combined in the 15 ml conical tube and then centrifuged for 10 min at 4°C and 400 x g. The remaining cell pellet was resuspended in 1 ml FACS buffer for further processing.

Alveolar macrophages are the predominant myeloid cell population in the BAL with no or barely any other macrophages being present.

Enzymatic tissue digestion

After BAL has been performed, the lungs were excised and transferred to a 1.5ml or 2 ml reaction tube containing 500 µl digestion buffer. The tissue was then homogenized using narrow scissors, transferred to a 60 mm cell culture dish containing 5ml digestion buffer and incubated at 37°C for 30min with occasional shaking. After incubation, the sample was passed through a 70 µm cell strainer into a 50 ml conical tube by using a syringe plunger, rinsed with PBS to a final volume of approximately 20 ml, and then centrifuged at 400 x g and 4°C for 5 min. The supernatant was discarded, and the remaining cell pellet was resuspended in 500 µl ACK buffer. After 3 min, 5 ml FACS buffer were added to stop erythrocyte lysis and another centrifugation was performed for 10 min at 400 x g and 4°C.

The supernatant was discarded, the cell pellet was resuspended in 500 µl FACS buffer and filtered through a 70 µm cell strainer.

IM are analyzed in enzymatically digested lung tissue. Since the preceding BAL does not remove all AM, their number will be reduced in enzymatically digested lung compared to lungs without lavage, but they will still be present.

Buffers

**FACS-buffer**	2 %	Fetal calf serum (FCS)
	0.02 %	Sodium azide
	in PBS	
**Digestion buffer (Lung)**	2mM	Sodium chloride ($MgCl_2$)
-	2mM	Calcium chloride ($CaCl_2$)
-	0.03 mg/ml	DNase I (Roche Diagnostics, Penzberg, Germany)
-	0.05 U/ml	Collagenase D (Roche Diagnostics, Penzberg, Germany)
	in HBSS	
**ACK buffer**	150 mM	Ammonium chloride (NH_4Cl)
	10 mM	Potassium hydrogen carbonate ($KHCO_3$)
		0.1 mM Di-sodium EDTA (Na_2-EDTA)

References:

[76] Cossarizza, A.; Chang, H.-D.; Radbruch, A.; Andrä, I.; Annunziato, F.; Bacher, P.; Barnaba, V.; Battistini, L.; Bauer, W.M.; Baumgart, S.; et al. Guidelines for the use of flow cytometry and cell sorting in immunological studies. *Eur. J. Immunol.* **2017**, *47*, 1584–1797, doi:10.1002/eji.201646632.

[77] Van Hoecke, L.; Job, E.R.; Saelens, X.; Roose, K. Bronchoalveolar Lavage of Murine Lungs to Analyze Inflammatory Cell Infiltration. *J. Vis. Exp.* **2017**, doi:10.3791/55398.

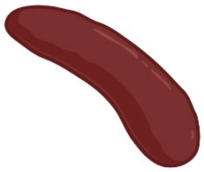

Preparation of macrophages from mouse spleen

(Adapted from a protocol by Fujiyama et al. [78])

Mice were euthanized and perfused with 10-15 ml cold PBS via the left ventricle. The spleen was removed and transferred into a 2 ml reaction vessel containing 1ml digestion buffer and homogenized using narrow scissors. Digestion buffer was added to a final volume of 2 ml and the homogenized tissue was then incubated for 30 min at 37°C with occasional gentle shaking. Subsequently, the homogenized tissue was passed through a 70 µm cell strainer into a 50 ml conical tube using a syringe plunger and rinsed with DMEM medium to a total volume of approx. 10 ml. Thereafter, the cell suspension was centrifuged for 5 min at 450 x g and 10°C. The supernatant was discarded and cells were resuspended in 5 ml of ACK buffer to lyse the erythrocytes. After incubation for 5 min at room temperature, lysis was stopped by addition of 25 ml PBS. The cell suspension was filtered through a 70 µm cell strainer and rinsed with FACS buffer to a final volume of approx. 35 ml. The sample was centrifuged for 5 min at 450 x g and 10°C, the supernatant was discarded and the cells were resuspended in FACS buffer.

Buffers

**Digestion buffer (Spleen)**	0.2 U/ml	Collagenase D (Roche Diagnostics, Penzberg, Germany)
	0,1 mg/ml	DNase I (Roche Diagnostics, Penzberg, Germany)
	0.6 U/ml	Dispase® (Roche Diagnostics, Penzberg, Germany)
		in DMEM medium with 2 % Fetal calf serum (FCS)
**ACK buffer**	150 mM	Ammonium chloride (NH_4Cl)
	10 mM	Potassium hydrogen carbonate ($KHCO_3$)
	0.1 mM	Di-sodium EDTA (Na_2-EDTA)
**FACS-buffer**	2 %	FCS
	0.02 %	Sodium azide
	in PBS	

Reference

[78] Fujiyama, S.; Nakahashi-Oda, C.; Abe, F.; Wang, Y.; Sato, K.; Shibuya, A. Identification and isolation of splenic tissue-resident macrophage sub-populations by flow cytometry. *Int. Immunol.* **2019**, *31*, 51–56, doi:10.1093/intimm/dxy064.

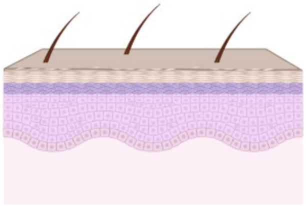

Preparation of Langerhans cells and Dermal Macrophages from mouse skin (ears)

(Adapted from a protocol by Cossarizza et al. [76])

Mice were euthanized, perfused with 10-15 ml cold PBS via the left ventricle and the ears were cut off close to the skull. 3 ml of *digestion buffer 1* were placed in one well of a 6-well plate. The ears were split at the cut edge into dorsal and ventral halves with the help of forceps, so that the inner dermal layer is exposed. Both halves were put to the solution with the dermal side facing down and incubated for at least 90 min at 37°C with occasional gentle swirling. Subsequently, the ear halves, dermis facing down, were transferred into an empty well and the epidermis was carefully scraped off with a flat spatula. Two 2 ml reaction vessels were prepared with 0.5 ml *digestion buffer 2* each. The separated epidermis and dermis were transferred into the reaction vessels and homogenized with narrow scissors. Vessels were filled up to 2 ml with *digestion buffer 2* and incubated for 60 min at 37°C with occasional shaking. Subsequently, the suspensions were passed through 70 µm cell strainers into separate 50 ml conical tubes for dermis and epidermis, respectively, using a syringe plunger and rinsed with RPMI 1640 medium to a total volume of approx. 10 ml, respectively. After centrifugation for 5 min at 450 x g and 10°C supernatants were discarded and the cells were resuspended in FACS buffer.

The Langerhans cells were analyzed using the epidermal cell preparation, dermal macrophages in the dermis preparation.

Tissue-specific cells are significantly enriched by this procedure, but some cells from each tissue type are often found also within the respective other one, since tissues are not completely segregated during mechanical separation by scraping.

Buffers

**Digestion buffer (Skin) 1**	2 U/ml	Dispase® (Roche Diagnostics, Penzberg, Germany)
	in RPMI 1640 medium with 10 % Fetal calf serum (FCS)	
**Digestion buffer (Skin) 2**	0.7 mg/ml	Collagenase D (Roche Diagnostics, Penzberg, Germany)
	0.03 mg/ml	DNase I (Roche Diagnostics, Penzberg, Germany)
	in RPMI 1640 medium with 10 % FCS	
**FACS-buffer**	2 %	FCS
	0.02 %	Sodium azide
	in PBS	

Reference

[76] Cossarizza, A.; Chang, H.-D.; Radbruch, A.; Andrä, I.; Annunziato, F.; Bacher, P.; Barnaba, V.; Battistini, L.; Bauer, W.M.; Baumgart, S.; et al. Guidelines for the use of flow cytometry and cell sorting in immunological studies. *Eur. J. Immunol.* **2017**, *47*, 1584–1797, doi:10.1002/eji.201646632.

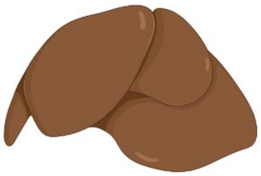

Preparation of Kupffer cells from mouse liver

Mice were euthanized and perfused with 10-15 ml cold PBS via the left ventricle. The liver was removed and transferred to a 2ml reaction tube containing 500 µl digestion buffer. The tissue was homogenized using narrow scissors and transferred to a 60 mm cell culture dish containing additional 5 ml of digestion buffer and incubated at 37°C for 30 min with occasional gentle shaking. After incubation, the sample was passed through a 70 µm cell strainer into a fresh 50 ml conical tube by using a syringe plunger and rinsed with PBS to a total volume of 25ml. The cell suspension was centrifuged at 18 x g and 4°C for 4 min ("Low-G-Centrifugation") and the supernatant was then carefully collected in a fresh 50ml conical tube. In this process, cells of the uppermost layer of the hepatocyte pellet (about 1 mm) were also transferred and the remaining cell pellet was discarded. Subsequently, the sample was centrifuged at 500 x g and 4°C for 5 min. The supernatant was discarded, starting with the yellowish fatty streaks on the surface of in order to avoid them flowing down the vessel wall during emptying. The cell pellet was resuspended in 5 ml of FACS buffer, transferred to a new tube and centrifuged again at 500 x g and 4°C for 5 min. The supernatant was discarded, and the cells were resuspended in 3 ml of FACS-Puffer for further processing.

Buffers

**Digestion buffer (Liver)**	2mM	Sodium chloride ($MgCl_2$)
-	2mM	Calcium chloride ($CaCl_2$)
-	0.03 mg/ml	DNase I (Roche Diagnostics, Penzberg, Germany)
-	0.05 mg/ml	Collagenase D (Roche Diagnostics, Penzberg, Germany)
	in HBSS	
**FACS-buffer**	2 %	Fetal calf serum (FCS)
	0.02 %	Sodium azide
	in PBS	

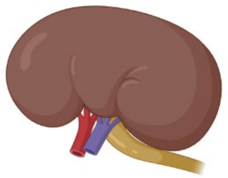

Preparation of murine Kidney-Resident Macrophages.

(Adapted from a protocol by Rubio-Navarro et al. [79])

Mice:

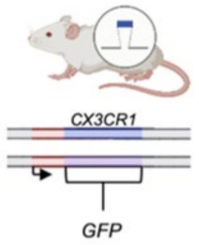

Since Kidney Resident MΦ are known to reveal high expression of CX3CR1 we used heterozygous B6 *cx3cr1*$^{+/gfp}$ mice which from one allele express Green Fluorescent Protein (GFP) under control of the *cx3cr1*-promoter and wild type CX3CR1 from the other allele.

Based on the previously described expression of CX3CR1 on renal resident macrophages [24], B6 *cx3cr1*$^{+/gfp}$ mice were used for this preparation. Mice were euthanized and perfused with 10-15 ml of cold PBS via the left ventricle. The kidneys were removed and transferred to a 1.5 ml reaction tube. The sample was homogenized using narrow scissors and incubated for 30min at 37°C with occasional gentle shaking. After incubation, the homogenized samples were passed through a 40 µm cell strainer using a syringe plunger, transferred to a new reaction tube and rinsed with PBS to a total volume of 10ml. Subsequently, centrifugation was performed at 400 x g and 4°C for 15 min. The supernatant was discarded, and the remaining cell pellet was incubated in 1ml ACK buffer. Lysis of erythrocytes was stopped after 90 s using 10 ml of FACS buffer and another centrifugation was performed for 10 min at 400 x g and 4°C. The supernatant was discarded afterwards, and the cells were resuspended in 500 µl FACS buffer and filtered through a 40 µm cell strainer.

Buffers

**Digestion buffer (Kidney)**	0.1 U/ml	Collagenase D (Roche Diagnostics, Penzberg, Germany)
	2 %	Fetal calf serum (FCS)
	in PBS	
**ACK buffer**	150 mM	Ammonium chloride (NH_4Cl)
	10 mM	Potassium hydrogen carbonate ($KHCO_3$)
	0.1 mM	Di-sodium EDTA (Na_2-EDTA)
**FACS-buffer**	2 %	FCS
	0.02 %	Sodium azide
	in PBS	

References:

[24] Yona, S.; Kim, K.-W.; Wolf, Y.; Mildner, A.; Varol, D.; Breker, M.; Strauss-Ayali, D.; Viukov, S.; Guilliams, M.; Misharin, A.; et al. Fate Mapping Reveals Origins and Dynamics of Monocytes and Tissue Macrophages under Homeostasis. *Immunity* **2013**, *38*, 79–91, doi:10.1016/j.immuni.2012.12.001.

[79] Rubio-Navarro, A.; Guerrero-Hue, M.; Martín-Fernandez, B.; Cortegano, I.; Olivares-Alvaro, E.; de las Heras, N.; Alía, M.; de Andrés, B.; Gaspar, M.L.; Egido, J.; et al. Phenotypic Characterization of Macrophages from Rat Kidney by Flow Cytometry. *J. Vis. Exp.* **2016**, *2016*, doi:10.3791/54599.

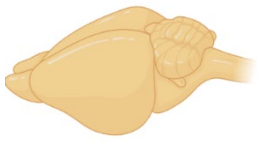

Preparation of microglia from mouse brain

(adapted from a protocol by Lee & Tansey) [81]

Mice:

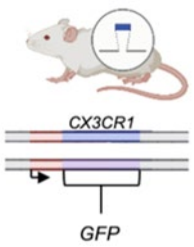

Since microglia are known to reveal high expression of CX3CR1 [80] we used heterozygous B6 *cx3cr1*$^{+/gfp}$ mice which on one allele express Green Fluorescent Protein (GFP) under control of the *cx3cr1*-promoter and wild type CX3CR1 on the other allele.

Mice were euthanized with isoflurane and perfused with 10-15 ml cold PBS via the left ventricle. Subsequently, the skull was opened with the aid of bone scissors, the brain was removed with the aid of a spoon-shaped spatula and transferred into a 5ml reaction vessel containing 1 ml digestion solution. Then the brain was homogenized using narrow scissors, the vessel was filled to 3 ml with digestion solution and incubated for 30 min at 37°C with occasional gentle shaking. Subsequently, the mixture was transferred into a 15 ml conical tube, 5 ml inactivation buffer were added and the homogenized tissue was centrifuged for 5 min at 250 x g and 10°C. The supernatant was discarded; the pellet was resuspended in 5 ml serum-free DMEM. The suspension was centrifuged once more for 5 min at 250 x g and 10°C. The pellet was again resuspended in 5 ml serum-free DMEM. The suspension was strained through a 40 µm cell strainer into a 50 ml conical tube using a syringe plunger, and rinsed with DMEM to a total volume of approx. 15 ml. After centrifugation for 4 min at 250 x g and 10°C the supernatant was discarded and the cell pellet was resuspended in 8 ml 37 % Percoll solution. This suspension was transferred into a 15 ml conical tube and underlaid with 4 ml of 70 % Percoll solution using a serological pipette. It was subsequently overlaid with 2 ml of HBSS and centrifuged for 40 min at 300 x g and 18°C without brake. The myelin-containing upper phase was discarded and 3 ml of the lower phase were collected in a separate 15 ml reaction tube. 9 ml of HBSS were added and the tube was centrifuged for 7 min at 500 x g and 4°C. The supernatant was discarded

carefully, so that the barely visible pellet remained intact. The cell pellet was then resuspended in 1 ml HBSS and transferred to a 1.5 ml reaction vessel. The vessel was centrifuged for 5 min at 800 x g at room temperature, the supernatant was discarded and the pellet was resuspended in FACS buffer.

Buffers

**Digestion buffer (Brain)**	0.1 mg/ml	DNase I (Roche Diagnostics, Penzberg, Germany)
	0.6 U/ml	Dispase® (Roche Diagnostics, Penzberg, Germany)
	in DMEM medium with 10 % Fetal calf serum (FCS)	
**Inactivation buffer**	10 mM	EDTA
	in DMEM medium with 10 % FCS.	
**Percoll stock solution**	13.5 ml	Percoll Plus (GE Healthcare, Uppsala, Sweden)
	1.5 ml	10x PBS
**Percoll 37%**	3.7 ml	Percoll stock solution
	5.3 ml	1x HBSS
**Percoll 70%**	7 ml	Percoll stock solution
	2 ml	1x HBSS
**FACS-buffer**	2 %	FCS
	0.02 %	Sodium azide
	in PBS	

Reference

[80] Jung, S.; Aliberti, J.; Graemmel, P.; Sunshine, M.J.; Kreutzberg, G.W.; Sher, A.; Littman, D.R. Analysis of fractalkine receptor CX(3)CR1 function by targeted deletion and green fluorescent protein reporter gene insertion. *Mol. Cell. Biol.* **2000**, *20*, 4106–14, doi:10.1128/MCB.20.11.4106-4114.2000.

[81] Lee, J.-K.; Tansey, M.G. Microglia isolation from adult mouse brain. *Methods Mol. Biol.* **2013**, *1041*, 17–23, doi:10.1007/978-1-62703-520-0_3.
