# Supplementary material for: There Is Strength in Numbers: Quantitation of Fc Gamma Receptors on Murine Tissue-Resident Macrophages
Source: Int J Mol Sci. 2021 Nov 10;22(22):12172. doi: 10.3390/ijms222212172 (PMC8620503; doi:10.3390/ijms222212172)
Supplement: Supplementary file 1 [file ijms-22-12172-s001.zip › Supplementary Table S1.pdf]

Supplementary Table S1: List of fluorochrome-conjugated antibodies used in this work.

antigen	clone	isotype	fluorochrome	final dilution [1:x]	manufacturer
CD3e	145-2C11	Arm. hamster IgG1 k	Pacific Blue™	100	Biolegend
CD11b	M1/70	rat IgG2b k	PerCP-Cy5.5	1000	Biolegend
CD11c	N418	Arm. hamster IgG	PE-Cy7	400	Biolegend
CD11c	HL3	Arm. Hamster	APC	300	BD Biosciences
CD16 (FcγRIII)	S17014E	rat IgG2a k	PE	10	Biolegend
CD16.2 (FcγRIV)	9E9	Arm. hamster IgG	PE	80	Biolegend
CD19	6D5	rat IgG2a k	Pacific Blue™	100	Biolegend
CD32b (FcγRIIb)	AT130.2	mouse IgG2a k	PE	10	Invitrogen
CD45	30-F11	rat IgG2b k	Brilliant Violet 510™	300	Biolegend
CD45	A20	mouse IgG2a k	Brilliant Violet 510™	300	BioLegend
CD64 (FcγRI)	X54-5/7.1	mouse IgG1 k	PE	10	Biolegend
CD86	GL-1	rat IgG2a k	FITC	100	BioLegend
CD102	3C4	rat IgG2a k	Alexa Fluor® 488	100	BioLegend
CD169	3D6.112	rat IgG2a k	PE-Cy7	100	Biolegend
CD206	C068C2	rat IgG2a k	Alexa Fluor® 647	100	BioLegend
CD207 (Langerin)	4C7	mouse IgG2a k	APC	100	Biolegend
F4/80	BM8	rat IgG2a	APC/Fire750™	100	Biolegend
F4/80	BM8	rat IgG2a	PE-Cy7	100	Biolegend
Gr-1	RB6-8C5	rat IgG2a k	Pacific Blue™	400	BioLegend
Ly6C	HK1.4	rat IgG2a k	APC	200	BioLegend
Ly6G	1A8	rat IgG2a	Alexa Fluor® 488	1000	Biolegend
MerTK	REA477	rec. hIgG1	PE-Cy7	200	BioLegend
MHC II I-A/I-E	M5/114.15.2	rat IgG2b k	Alexa Fluor® 647	200	Biolegend
MHC II I-A^b	AF6-120.1	mouse IgG2a k	FITC	300	BD Biosciences
Siglec-F	S17007L	rat IgG2a k	KIRAVIA Blue 520™	200	BioLegend
SIGN-R1	22D1	Arm. hamster	Alexa Fluor® 647	50	eBioscience
Tim4	F31-5G3	rat IgG2a k	Alexa Fluor® 647	100	BioLegend
